# SPICE-ML Algorithm for Direction-of-Arrival Estimation

**DOI:** 10.3390/s20010119

**Published:** 2019-12-24

**Authors:** Yu Zheng, Lutao Liu, Xudong Yang

**Affiliations:** 1 College of Information and Communication Engineering, Harbin Engineering University, Harbin 150001, China; yu_zheng78@163.com (Y.Z.); 06170817@hrbeu.edu.cn (X.Y.); 2 The 14th Research Institute Electronics Technology Group Corporation, Nanjing 210000, China

**Keywords:** direction-of-arrival estimation, covariance fitting criterion, sparse iterative covariance-based estimation approach, maximum likelihood estimation

## Abstract

Sparse iterative covariance-based estimation, an iterative direction-of-arrival approach based on covariance fitting criterion, can simultaneously estimate the angle and power of incident signal. However, the signal power estimated by sparse iterative covariance-based estimation approach is inaccurate, and the estimation performance is limited to direction grid. To solve the problem above, an algorithm combing the sparse iterative covariance-based estimation approach and maximum likelihood estimation is proposed. The signal power estimated by sparse iterative covariance-based estimation approach is corrected by a new iterative process based on the asymptotically minimum variance criterion. In addition, a refinement procedure is derived by minimizing a maximum likelihood function to overcome the estimation accuracy limitation imposed by direction grid. Simulation results verify the effectiveness of the proposed algorithm. Compared with sparse iterative covariance-based estimation approach, the proposed algorithm can achieve more accurate signal power and improved estimation performance.

## 1. Introduction

Direction-of-arrival (DOA) refers to the process of retrieving the direction information of several electromagnetic waves from the outputs of a number of receiving antennas that form a sensor array. DOA estimation is a major topic in array signal processing and plays a very important role in many applications, e.g., wireless communications, medical imaging, and radar systems [1,2,3],. Moreover, wireless sensor networks (WSNs) are an emerging paradigm in wireless communications [4]. In WSNs one of the most significant challenges is localization in which DOA is a major parameter to estimate [5,6]. The research on DOA estimation is mainly divided into two aspects: (1) array geometry, such as the recently proposed nested array [7] and coprime array [8,9]. (2) DOA estimation algorithm with higher estimation accuracy. In general, the algorithm applied to DOA estimation may be classified into three parts: nonparametric, parametric, and semiparametric [10].

A main example of the nonparametric methods is delay-and-sum (DAS) beamformer [10] in which the received signal from each sensor are weighted and delayed so as to focus on different points in space. It is a data-independent estimation technique which is traditionally adopted due to its low computational burden and high-signal-to-noise ratio (SNR) properties. However, data-independent approaches suffer from leakage problem. The local leakage will reduce the resolution, which makes the DAS beamformer unable to distinguish the incident signals with close frequency components. Global leakage will lead to false alarms. Adaptive Capon beamformer can improve the DAS method, but it is limited to independent signal [11]. Parametric methods, especially subspace methods such as multiple signal classification (MUSIC) [12], estimation of signal parameters via rotational invariance techniques (ESPRIT) [13], where parameters are estimated by studying the subspace of the data covariance. They can provide higher resolution than nonparametric methods, but adimit two shortcomings: (1) they require the knowledge of the number of source, and (2) work well only when the sources are independent. Recently, these two kinds of approaches have been combined, leading to the so-called semiparametric approache. The recently developed iterative adaptive algorithm (IAA) [14] largely eliminates the leakage problem of the beamformer and is robust to coherent sources. However, the IAA algorithm still has resolution limitations, especially at low SNR situations. Stoica et al. have recently proposed a user parameter-free sparse iterative covariance-based estimation (SPICE) approach in [15,16], based on minimizing a covariance fitting criterion. SPICE is able to provide excellent resolution and low sidelobe levels while maintaining robustness to coherent sources. Moreover, the power of the signal can be estimated simultaneously. However, as with most power-based sparse methods, the estimation accuracy of the SPICE algorithm is limited to the direction grid. In addition, the signal power estimated by SPICE is inaccurate, especially in the case of coherent signal. In [17], a method named likelihood-based estimation of sparse parameters (LIKES) is proposed, where the same covariance fitting criterion is adopted as SPICE algorithm. The difference is that the weights in LIKES are adaptive rather than constant in SPICE. Compared with the SPICE algorithm, LIKES can obtain more accurate DOA estimation, but at the expense of computational cost. The DOA estimation accuracy will be limited to direction grid because the spatial domain is also discretized in LIKES algorithm. In [18], an iterative algorithm based on the combination of Capon and SPICE (C-SPICE) is proposed, and a mobile average initialization technology is introduced to realize DOA estimation by using the spatial spectrum information estimated in the previous snapshot in the next snapshot.

DAS has low computational burden and accurate signal power estimation, but low resolution. The resolution of IAA is improved, but still not high enough to distinguish two signals which are close to each other. SPICE has high resolution, but the signal power estimated by SPICE is not accurate, and the DOA estimation accuracy is limited to the direction grid. In order to obtain an algorithm with high resolution, accurate signal power estimation and high estimation accuracy, we propose a SPICE and maximum likelihood (ML) estimation approach (SPICE-ML) in this paper. An iterative correction process is derived through the asymptotically minimum variance (AMV) criterion [19,20] so that the power of the signal estimated are more accurate. Moreover, a maximum likelihood cost function is used to refine the DOA estimation, thereby combating the limit of estimation accuracy caused by the direction grid. Numerical simulations are designed to show the effectiveness and superiority in estimation accuracy of the proposed algorithm.

This paper is organized as follows. The signal model for DOA estimation is introduced in Section 2 for far-field, narrowband sources. The concept of sparse representation is also discussed in Section 2. The SPICE algorithm is introduced in Section 3, including the covariance fitting criterion and updating formulas. Section 4 is the main highlight of this paper in which the SPICE-ML is proposed. Compared with SPICE algorithm, SPICE-ML can receive more accurate signal power estimation and DOA estimation accuracy. Numerical simulations are presented in Section 5, and the conclusion and some future research directions are drawn in Section 6.

Notations: Vectors, matrices are denoted by boldface lowercase letters and boldface uppercase letters, respectively. ${\left(\cdot \right)}^{T}$, ${\left(\cdot \right)}^{*}$, ${\left(\cdot \right)}^{-1}$ and ${\left(\cdot \right)}^{H}$ represent the transpose, the conjugate, the inverse and the complex conjugate transpose of vectors or matrices. ⊗ is the Kronecker product and $\mathrm{tr}\left(\cdot \right)$ represents the trace of a matrix. $E\left[\cdot \right]$, $\det\left(\cdot \right)$ and $\mathrm{vec}\left(\cdot \right)$ denote the operator of expectation, determinant and vectorization. $∥\cdot ∥$ denotes the Euclidean norm for vectors and the Frobenius norm for matrices.

## 2. Signal Model

**Figure 1:**
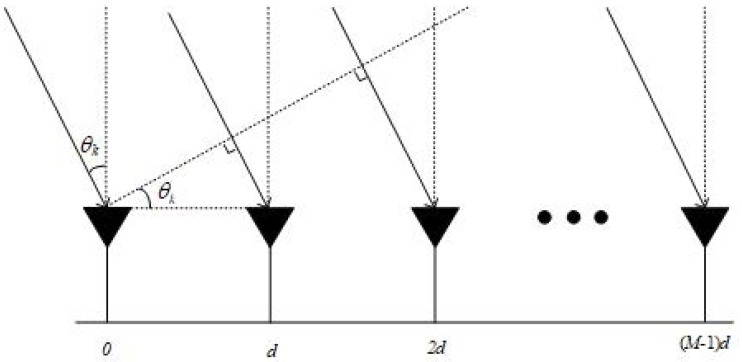
Signal model.

Assume *N* far-field, uncorrelated narrowband signals from directions $\Omega =\left[\theta_{1},\ldots \theta_{N}\right]$ impinging on an uniform line array (ULA), the number of ULA is *M* and the inter-element spacing equals to a half of the signal wavelength. Let ${\left\{\theta_{k}\right\}}_{k=1}^{K}$ denote a direction grid that covers Ω, where K≫N, $\theta_{k}\in \left[-90^{\circ }\right.\;\left.90^{\circ }\right)$. Assume the direction gird is fine enough so that the true location parameters of the sources lie on (or, practically, close to) the grid. The signal model is shown in Figure 1.

Then the output of the array can be modeled as:

(1) $$x\left(t\right)=\sum_{k=1}^{K}a\left(\theta_{k}\right)s_{k}\left(t\right)+n\left(t\right)=A\left(\theta \right)S\left(t\right)+n\left(t\right),\;\;\;t=1,\ldots ,T$$

where $A\left(\theta \right)=\left[a\left(\theta_{1}\right),a\left(\theta_{2}\right),\ldots ,a\left(\theta_{K}\right)\right]$ denotes the array manifold matrix and $a\left(\theta_{k}\right)$ is the steering vector corresponding to $\theta_{k}$. *T* denotes the number of snapshots. $S\left(t\right)={\left[s_{1}\left(t\right),s_{2}\left(t\right),\ldots ,s_{K}\left(t\right)\right]}^{T}$ contains the *K* unknown complex-valued signals, and $n\left(t\right)$ is the additional noise term. We assume that $E\left[n\left(t\right)n^{H}\left(t\right)\right]=\sigma I_{M}$, where σ is the power of noise, and $I_{M}$ is a M×M identical matrix. Let us further assume that $E\left(S\left(t\right)S^{H}\left(t\right)\right)=P_{k}I_{M}$, $P_{K}=\mathrm{Diag}\left(p_{1},p_{2},\ldots p_{K}\right)$, where $p_{k}$ represents the unknown signal power at $\theta_{k}$. Moreover the signal $S\left(t\right)$ and noise $n\left(t\right)$ are assumed to be statistically independent. Therefore, we have the covariance matrix of $x\left(t\right)$

(2) $$R=AP_{K}A^{H}+\sigma I_{M}.$$

In practice, this covariance matrix is usually estimated by the sample covariance matrix

(3) $$\hat{R}=\frac{1}{T}\sum_{t=1}^{T}x\left(t\right)x^{H}\left(t\right),$$

Note that only few sources exist in practice, therefore in the signal matrix

(4) $$S=\left[\begin{array}{lllll} \;s_{1}\left(1\right) & s_{1}\left(2\right) & \cdots & \cdots & s_{1}\left(T\right)\\ \;\;\vdots & \vdots & \vdots & \vdots & \vdots \\ \;s_{K}\left(1\right) & s_{K}\left(2\right) & \cdots & \cdots & s_{K}\left(T\right) \end{array}\right],$$

only a small number of rows are different from zeros. Thus, the DOA estimation problem is then changed to decide from $x\left(t\right)$ which rows of the signal matrix S are non-zero.

## 3. The SPICE Algorithm

The SPICE algorithm is derived from a robust covariance fitting criterion. It has a sound statistical foundation, it does not require any hyper-parameters, and yet it has global convergence properties. Moreover, the signal and noise power are estimated in a natural manner. Although the signal are assumed to be independent, the SPICE algorithm is still robust to coherent signal [15]. In this paper, we only consider the case in which T>M and the variances of noise are identical (see [15] for the case of T<M and noise with different variances).

Equation (2) can be rewrite as

(5) $$R=A_{aug}{\mathrm{PA}}_{aug}^{H},$$

where

(6) $$A_{aug}=\left[a\left(\theta_{1}\right)\;a\left(\theta_{2}\right)\;\ldots \;a\left(\theta_{K}\right)\;\;I_{M}\right]=\left[a_{1}\;a_{2}\;\ldots \;a_{K}\;a_{K+1}\;\;\ldots \;a_{K+M}\;\;\right],$$

(7) $$P=\left[\begin{array}{c} p_{1}\;\;0\;\;\cdots \;\;\cdots \;\;\;\cdots \;\;\cdots \;0\\ \;\vdots \;\;\;\;\vdots \;\;\;\ddots \;\;\;\vdots \;\;\;\;\;\vdots \;\;\;\;\vdots \;\;\vdots \\ \;0\;\;\cdots \;\;\cdots \;\;p_{K}\cdots \;\;\cdots \;0\\ \;0\;\;\cdots \;\;\cdots \;\;\cdots \;\;\;\sigma_{1}\cdots \;0\\ \;\vdots \;\;\;\;\vdots \;\;\;\;\;\vdots \;\;\;\;\vdots \;\;\;\;\;\vdots \;\;\;\ddots \vdots \\ \;0\;\;\cdots \;\;\cdots \;\;\cdots \;\;\cdots \;\;\cdots \sigma_{M} \end{array}\right]=\left[\begin{array}{c} p_{1}\;\;0\;\;\cdots \;\;\;\cdots \;\;\;\;\;\cdots \;\;\;\cdots \;\;0\\ \;\vdots \;\;\;\;0\;\;\;\ddots \;\;\;\vdots \;\;\;\;\;\;\;\;\vdots \;\;\;\;\;\vdots \;\;\;\vdots \\ \;0\;\;\cdots \;\;\cdots \;\;p_{K}\;\;\;\;\cdots \;\;\;\cdots \;\;0\\ \;0\;\;\cdots \;\;\cdots \;\;\cdots \;\;\;p_{K+1}\;\;\cdots \;\;0\\ \;\vdots \;\;\;\;\vdots \;\;\;\;\;\vdots \;\;\;\;\vdots \;\;\;\;\;\vdots \;\;\;\;\ddots \;\;\;\;\;\;\vdots \\ \;0\;\;\cdots \;\;\cdots \;\;\cdots \;\;\cdots \;\;\cdots \;\;p_{K+M} \end{array}\right].$$

The SPICE algorithm considers the following weighted covariance fitting criterion:

(8) $$f={∥R^{-1/2}\left(\hat{R}-R\right){\hat{R}}^{-1/2}∥}^{2},$$

a simple calculation shows that Equation (8) can be expressed as:

(9) $$\begin{array}{cc} f & =\mathrm{tr}\left[R^{-1}\left(\hat{R}-R\right){\hat{R}}^{-1}\left(\hat{R}-R\right)\right]\\ & =\mathrm{tr}\left[\left(R^{-1}\hat{R}-I\right)\left(I-R^{-1}\hat{R}\right)\right]\\ & =\mathrm{tr}\left(R^{-1}\hat{R}\right)+\mathrm{tr}\left({\hat{R}}^{-1}R\right)-2M \end{array},$$

where

(10) $$\mathrm{tr}\left({\hat{R}}^{-1}R\right)=\sum_{k=1}^{K+M}p_{k}a_{k}^{*}{\hat{R}}^{-1}a_{k}.$$

The minimization of *f* obtained from Equations (9) and (10) is equivalent to the minimization of the function

(11) $$g=\mathrm{tr}\left({\hat{R}}^{1/2}R^{-1}{\hat{R}}^{1/2}\right)+\sum_{k=1}^{K+M}\left(a_{k}^{*}{\hat{R}}^{-1}a_{k}\right)p_{k},$$

The minimization problem with respect to $p_{k}$ in Equation (11) is a semidefinite program (SDP) and therefore is a convex problem [21]. However, the calculation of Equation (11) as a SDP is very computationally intensive. It can be seen from Equation (10) that a consistent estimation $\mathrm{tr}\left({\hat{R}}^{-1}R\right)=M$ can be obtained when *T* tends to be infinite. Hence, the probem of minimizing *g* can be reformulated as the following constrained minimization problem:

(12) $$\min_{\left\{p_{k}\geq 0\right\}}\;\mathrm{tr}\left({\hat{R}}^{1/2}R^{-1}{\hat{R}}^{1/2}\right)\;\;s.t.\sum_{k=1}^{K+M}\omega_{k}p_{k}=1,$$

where

(13) $$\omega_{k}=a_{k}^{*}{\hat{R}}^{-1}a_{k}/M.$$

The minimization of the objective in Equation (12) can be solved by means of a cyclic algorithm [15], leading to the updated formulas of the SPICE algorithm

(14) $$p_{k}^{i+1}=p_{k}^{i}\frac{∥a_{k}^{*}R^{-1}\left(i\right){\hat{R}}^{1/2}∥}{\omega_{k}^{1/2}\rho \left(i\right)}\;\;\;\;\;k=1,\ldots ,K,$$

(15) $$\rho \left(i\right)=\sum_{k=1}^{K}\omega_{k}^{1/2}p_{k}^{i}∥a_{k}^{*}R^{-1}\left(i\right){\hat{R}}^{1/2}∥+\gamma^{1/2}\sigma^{i}∥R^{-1}\left(i\right){\hat{R}}^{1/2}∥,$$

(16) $$\sigma^{i+1}=\sigma^{i}\frac{∥R^{-1}\left(i\right){\hat{R}}^{1/2}∥}{\gamma^{1/2}\rho \left(i\right)},\gamma =\sum_{k=K+1}^{K+M}\omega_{k}.$$

where index *i* denotes the number of iteration. The algorithm can be initialized by means of the DAS method. The cyclic operation of the cyclic algorithm makes the objective function in Formula (12) monotonically decrease, and the minimization process is a convex problem. Therefore, the result of SPICE algorithm has global convergence. In [22], it is proved that the limit point of the iterative process of SPICE is the global solution of the minimization problem (12) under the weak condition where $p_{k}^{0}>0$ and R(i)≥0 in each iteration.

## 4. The SPICE-ML Algorithm

SPICE algorithm has a bias in estimating the signal power, especially in the case of coherent sources (as shown in the simulation part). To solve this problem, an iteration calibration process of signal power is obtained by using AMV criterion. After applying the vectorization operator to the matrix R, we can receive the vector $r\left(p\right)$

(17) $$r\left(p\right)=\mathrm{vec}\left(R\right)=\tilde{A}p,$$

where $\tilde{A}=\left[{\tilde{A}}_{1},{\bar{a}}_{K+1}\right],{\tilde{A}}_{1}=\left[{\bar{a}}_{1},\ldots ,{\bar{a}}_{K}\right],{\bar{a}}_{k}=a_{k}^{*}⊗a_{k},k=1,\ldots K.\;{\bar{a}}_{K+1}=\mathrm{vec}\left(I_{M}\right),p=\left[p_{1},\ldots ,p_{K}\right]$. Note that the Gaussian circular asymptotic covariance matrix $\hat{r}\overset{\mathrm{def}}{=}\mathrm{vec}\left(\hat{R}\right)$ is give by [20]

(18) $$C_{r}=R^{*}⊗R.$$

Suppose that p can be identifiable from $r\left(p\right)$. According to [19,20], it can be proved that the covariance matrix ${\mathrm{Cov}}_{p}^{\mathrm{Alg}}$ is bounded below by the following real symmetric positive definite matrix

(19) $${\mathrm{Cov}}_{p}^{\mathrm{Alg}}\geq {\left[{\tilde{A}}_{d}^{H}C_{r}^{-1}{\tilde{A}}_{d}\right]}^{-1},$$

where ${\tilde{A}}_{d}=dr\left(p\right)/dp$, $d\left(\cdot \right)/d\left(\cdot \right)$ is the operation of differential. In addition, we can obtain this lower bound by minimizing the following AMV criterion

(20) $$\begin{array}{c} \hat{p}=\arg\;\min_{p}\;f\left(p\right)\\ f\left(p\right)\overset{\mathrm{def}}{=}{\left[\hat{r}-r\left(p\right)\right]}^{H}C_{r}^{-1}\left[\hat{r}-r\left(p\right)\right]. \end{array}$$

From Equations (18) and (20), the estimation of p and σ are given by the following iterative s

(21) $${\hat{p}}_{k}^{\left(i+1\right)}=\frac{a_{k}^{H}R^{-1\left(i\right)}\hat{R}R^{-1\left(i\right)}a_{k}}{{\left(a_{k}^{H}R^{-1\left(i\right)}a_{k}\right)}^{2}}+p_{k}^{\left(i\right)}-\frac{1}{a_{k}^{H}R^{-1\left(i\right)}a_{k}},k=1,\ldots K,$$

(22) $${\hat{\sigma }}^{\left(i+1\right)}=\left(\mathrm{tr}\left(R^{-2\left(i\right)}\hat{R}\right)+{\hat{\sigma }}^{\left(i\right)}\mathrm{tr}\left(R^{-2\left(i\right)}\right)-\mathrm{tr}\left(R^{-1\left(i\right)}\right)\right)/\mathrm{tr}\left(R^{-2\left(i\right)}\right),$$

where $R^{\left(i\right)}=AP^{\left(i\right)}A^{H}+{\hat{\sigma }}^{\left(i\right)}I_{M}$, $P^{\left(i\right)}=\mathrm{Diag}\left({\hat{p}}_{1}^{\left(i\right)},\ldots ,{\hat{p}}_{K}^{\left(i\right)}\right)$. The initialization of ${\left\{{\hat{p}}_{k}^{\left(0\right)}\right\}}_{k=1}^{K},{\hat{\sigma }}^{\left(0\right)}$ is provided by the SPICE algorithm. In practice the noise power $\sigma_{k},k=1,\ldots M$ estimated by the SPICE algorithm may be different. [23] shows that the degradation of accuracy comapared with that achieved by imposing $\sigma_{k}^{2}\neq \sigma^{2},\forall k$, is not significant. Through the iterative Formula (21), the signal power are continuously corrected.

The estimation accuracy of the SPICE algorithm is limited to the fineness of the direction grid. A coarse grid would lead to the degradation of estimation accuracy and a problem of computational complexity arises if a high-density grid is employed. Therefore, how to choose the direction grid becomes a difficult problem. In order to overcome the limit of estimation accuracy caused by the direction grid, the DOA estimation $\hat{\Omega }=\left({\hat{\theta }}_{1}\;\ldots \;{\hat{\theta }}_{N}\right)$ is refined by iteratively minimizing a stochastic ML cost function.

The stochastic negative log-likelihood function of $x\left(t\right)$ can be expressed as [24]

(23) $$L\left(\theta \right)=\ln\left(\det\left(R\right)\right)+\mathrm{tr}\left(R^{-1}\hat{R}\right).$$

The covariance matrix of noise is defined as

(24) $$Q_{k}=R-p_{k}a_{k}a_{k}^{H},\;\;k=1,\ldots ,K.$$

Applying matrix inverse lemma to Formula (24)

(25) $$R^{-1}=Q_{k}^{-1}-p_{k}\beta_{k}b_{k}b_{k}^{H},\;\;k=1,\ldots ,K.$$

where $b_{k}=Q_{k}^{-1}a_{k}$, $\beta_{k}={\left(1+p_{k}a_{k}^{H}Q_{k}^{-1}a_{k}\right)}^{-1}$.

Besides,

(26) $$\mathrm{tr}\left(R^{-1}\hat{R}\right)=\mathrm{tr}\left(Q^{-1}\hat{R}\right)-p_{k}\beta_{k}b_{k}^{H}\hat{R}b_{k}.$$

Then use the algebraic identity $\det\left(I+\mathrm{AB}\right)=\det\left(I+\mathrm{BA}\right)$ and obtain

(27) $$\begin{array}{cc} \ln\left(\det\left(R\right)\right) & =\ln\left(\det\left(Q_{k}+p_{k}a_{k}a_{k}^{H}\right)\right)=\ln\left[\left(1+p_{k}a_{k}^{H}Q_{k}^{-1}a_{k}\right)\det\left(Q_{k}\right)\right]\\ & =\ln\left(\det\left(Q_{k}\right)\right)-\ln\left(\beta_{k}\right) \end{array}.$$

From Equations (23), (26) and (27), we can derive

(28) $$\begin{array}{cc} L\left(\theta \right) & =\ln\left(\det\left(Q_{k}\right)\right)+\mathrm{tr}\left(Q_{k}^{-1}\hat{R}\right)-\left(\ln\left(\beta_{k}\right)+p_{k}\beta_{k}\left(b_{k}^{H}\hat{R}b_{k}\right)\right)\\ & =L\left(\theta_{-k}\right)-l\left(\theta_{k}\right) \end{array},$$

(29) $$l\left(\theta_{k}\right)=\ln\left(\frac{1}{1+p_{k}\alpha_{1,k}}\right)+p_{k}\frac{\alpha_{2,k}^{N}}{1+p_{k}+\alpha_{1,k}},$$

where $\alpha_{1,k}=a_{k}^{H}Q_{k}^{-1}a_{k},\alpha_{2,k}^{N}={\left(a_{k}^{H}Q_{k}^{-1}{\hat{R}Q}_{k}^{-1}a_{k}\right)}^{-1},Q_{k}=R-p_{k}a_{k}a_{k}^{H},k=1,\ldots ,K.$

The ML cost function can be decomposed into two parts: $L\left(\theta_{-k}\right)$, the marginal likelihood function with parameter $\theta_{k}$ excluded, and $l\left(\theta_{k}\right)$ concerning $\theta_{k}$. Therefore, the ML cost function (23) with respect to $\theta_{k}$ is equivalent to the ML function (29). Although the calculation of multi-dimensional optimization problem is very complex, the minimization problem in Equation (29) can be effectively solved by using the Nelder–Mead algorithm [25]. Moreover, the Nelder–Mead algorithm has already been built into the “fminsearch” function in MATLAB. Suppose that we have obtained the corrective parameters ${\left\{{\hat{p}}_{k}^{\left(i\right)}\right\}}_{k=1}^{K},{\hat{\sigma }}^{\left(i\right)}$ and ${\left\{{\hat{\theta }}_{k}^{\left(0\right)}\right\}}_{k=1}^{N}$, then the refinement result can be achieved by minimizing Formula (29).

The SPICE-ML algorithm is summarized in Table 1.

## 5. Simulation Results

We evaluated the performance of the proposed SPICE-ML algorithm and compared it with DAS, IAA, and SPICE in this section. We employed a uniform linear array with 12 sensors, and the inter-element spacing was half-wavelength. The direction grid ${\left\{\theta_{k}\right\}}_{k=1}^{K}$ uniformly covered the entire DOA space $\Omega =\left[-90^{\circ }\right.\;\left.90^{\circ }\right)$ with a step size of 0.5°. The SNR was defined as

(30) $$\mathrm{SNR}=10\log_{10}\left(\frac{p_{avg}}{\sigma }\right)\;\left[\mathrm{dB}\right]$$

where $p_{avg}$ denotes the average power of the signal. The iteration termination condition is set to ${∥{\hat{p}}^{i+1}-{\hat{p}}^{i}∥}_{2}/{∥{\hat{p}}^{i}∥}_{2}<10^{-3}$.

**Figure 2:**
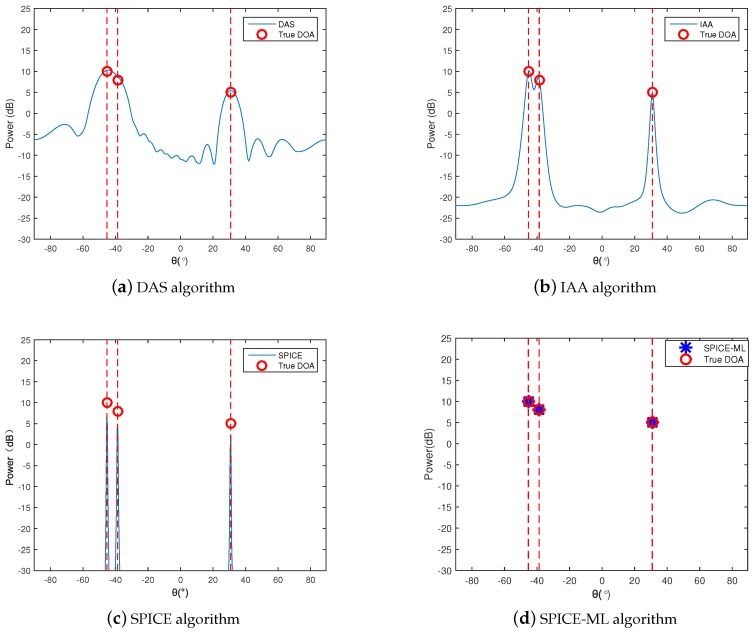
Root mean square error (RMSE) with independent signal.

**Figure 3:**
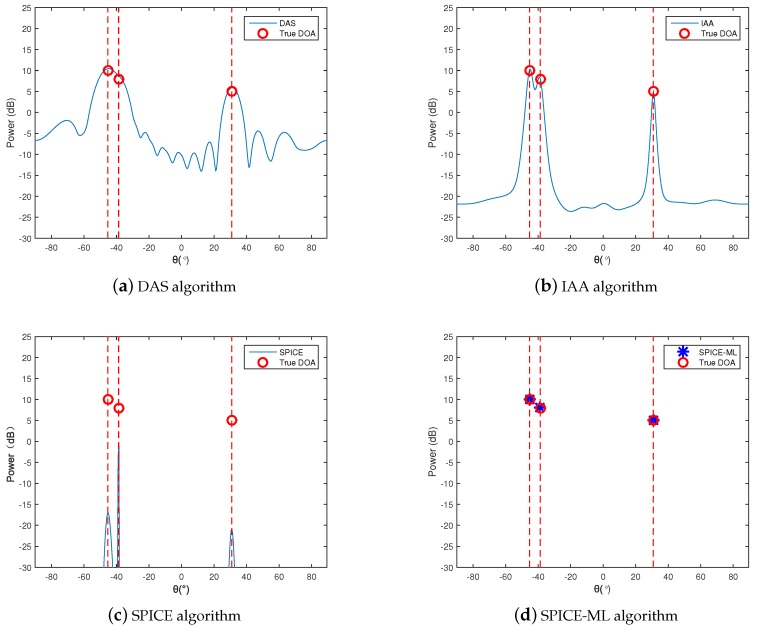
RMSE with coherent signal.

Three signals with power 10 dB, 8 dB, and 5 dB from $\theta_{1}=-45.3^{\circ },\theta_{2}=-38.7^{\circ },\theta_{3}=30.8^{\circ }$ impinged on the array. The additional noise was Gaussian white noise with equal power, and the SNR is set to 20 dB. The number of snapshots was given by T=50. Both independent and coherent sources are shown in Figure 2 and Figure 3. For the case of coherent sources, the signals at $\theta_{1}$ and $\theta_{3}$ shared the same phases but were independent to the signal at $\theta_{2}$.

It can be seen from the simulation results of Figure 2a and Figure 3a, the DAS method failed to seperate the two close sources at $\theta_{1}$ and $\theta_{2}$ owing to the smearing effects and limited resolution. The IAA algorithm could significantly reduce the smearing effect so that the sidelobes in Figure 2b and Figure 3b were lower, but the resolution was still not high. The SPICE algorithm was capable of resolving the three sources, but the signal power estimated was not accurate, especially in the case of coherent sources, as shown in Figure 2c and Figure 3c. The SPICE-ML algorithm proposed in this paper further corrected the signal power estimated by the SPICE algorithm, leading to a more accurate signal power estimation. Figure 2d and Figure 3d illustrate the effectiveness and high resolution of the proposed SPICE-ML algorithm.

**Figure 4:**
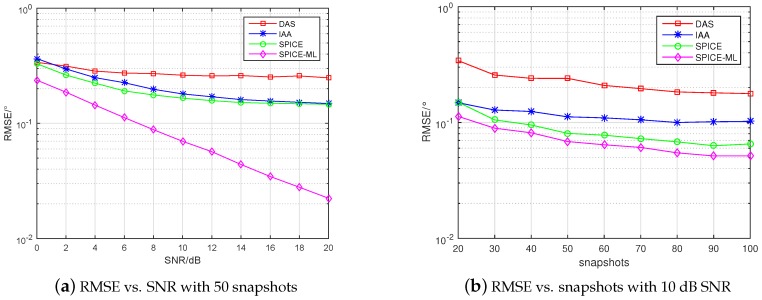
RMSE with independent signal.

**Figure 5:**
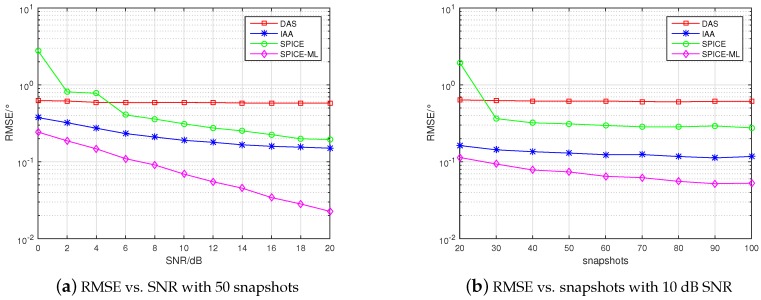
RMSE with coherent signal.

Next, we evaluate the root mean square error (RMSE) of DOA estimation through Monte-Carlo simulations. The defination of RMSE is:

(31) $$\mathrm{RMSE}=\sqrt{\frac{1}{LK}\sum_{l=1}^{L}\sum_{k=1}^{K}{\left({\hat{\theta }}_{k}{,}_{l}-\theta_{k}\right)}^{2}},$$

where ${\hat{\theta }}_{k}{,}_{l}$ is the estimated DOA of the *k* signal in the *l*-th Monte-Carlo trial, and *L* is the total number of Monte-Carlo trials. Assume that three signals are randomly located at $\left[-60^{\circ }\;\;60^{\circ }\right)$. Considering both independent and coherent sources, respectively. The number of Monte-Carlo trials is 500. The RMSE curves are shown in Figure 4 and Figure 5. Moreover, we display the values of RMSE at each SNR and snapshot in Table 2, Table 3, Table 4 and Table 5.

From Figure 4a,b, it can be seen that in the case of independent signal the RMSE of all algorithms decreased with the increase of SNR and snapshots, and the RMSE of SPICE-ML proposed in this paper was the lowest, which illustrates that the proposed SPICE-ML approach outperformed the other methods. From the Table 2 and Table 3, specifically, compared with SPICE, SPICE-ML could efficiently reduce the RMSE vs. SNR from 0.147°–0.330° to 0.022°–0.238°, the RMSE vs. snapshots from 0.065°–0.150° to 0.051°–0.113° in the case of independent signal. From Figure 5a,b, we can see the estimation accuracy of the SPICE algorithm declined dramatically in the case of coherent signal compared with that in the case of independent signal. However, source coherence did not degrade the DAS and IAA algorithm. Notably, SPICE-ML still offered the best estimation performance in the case of coherent signal, because the angle estimated by SPICE was further refined by minimizing an ML function. From the Table 4 and Table 5, we can see that compared with SPICE, the RMSE vs. SNR and snapshots were reduced from 0.195°–2.777° to 0.023°–0.244°, from 0.276°–1.950° to 0.053°–0.114°, respectively. We can also observe that there existed plateau effects for DAS, IAA, and SPICE. This is because all these methods estimate DOA by means of selecting one element from a fixed set of direction grid, but there always exists an estimation bias no matter how fine the direction grid is. Theoretically, bias can be reduced by selecting a dense gird. However, a dense grid will lead to large computation cost and is not applicable in practice. On the contrary, the SPICE-ML algorithm would not suffer from the plateau effect because of a refinement DOA estimation procedure based on minimizing a ML cost function.

## 6. Conclusions

In this paper, we combined the SPICE DOA estimation algorithm and maximum likelihood estimation and proposed a SPICE-ML algorithm. Compared with the SPICE algorithm, the SPICE-ML method derive an iterative correction procedure for signal power estimation based on the AMV criterion, and combat the limitation of the direction grid by minimazing a Maximum Likelihood cost function. The simulation results verify the effectiveness of the proposed algorithm. In addition, the superiority of the SPICE-ML algorithm in DOA estimation accuracy is illustrated compared with the DAS, IAA, and SPICE algorithms. Specifically, comparing with SPICE, SPICE-ML can efficiently reduce the RMSE vs. SNR from 0.147°–0.330° to 0.022°–0.238° in the case of independent signal, from 0.195°–2.777° to 0.023°–0.244° in coherent signal situation, the RMSE vs. snapshots from 0.065°–0.150° to 0.051°–0.113° in the case of independent signal, from 0.276°–1.950° to 0.053°–0.114° in coherent signal situation. In the future, it will be of interest to study the performance of SPICE-ML algorithm in the case of single snapshot. This paper only considers one-dimensional DOA estimation, so another future direction of interest would be the extension of SPICE-ML to two-dimensional DOA estimation.

## Figures and Tables

**Table 1:** The SPICE-ML algorithm.

**step1.** Initialization:${\left\{{\hat{p}}_{k}^{\left(0\right)}\right\}}_{k=1}^{K}$, ${\hat{\sigma }}^{\left(0\right)}$ and ${\left\{{\hat{\theta }}_{k}^{\left(0\right)}\right\}}_{k=1}^{N}$ are obtained from SPICE algorithm.
**repeat**
**step2.** Compute $R^{\left(i\right)},Q_{k}^{\left(i\right)}$.
**step3.** Update $p_{k}$ and σ according to Formula (21) and (22).
**step4.** Minimizing the ML cost function with respect to $\theta_{k}$ and obtain the refined estimated DOA ${\left\{{\hat{\theta }}_{k}\right\}}_{k=1}^{N}$.
**end for**
**until** (convergence)

**Table 2:** RMSE vs. signal to noise ratio (SNR) with independent signal.

SNR	0	2	4	6	8	10	12	14	16	18	20
DAS	0.339	0.315	0.285	0.274	0.271	0.262	0.258	0.260	0.253	0.258	0.249
IAA	0.365	0.297	0.250	0.226	0.198	0.180	0.170	0.161	0.156	0.153	0.149
SPICE	0.330	0.263	0.223	0.192	0.177	0.166	0.158	0.152	0.149	0.148	0.147
SPICE-ML	0.238	0.187	0.144	0.112	0.088	0.070	0.057	0.044	0.035	0.028	0.022

**Table 3:** RMSE vs. snapshots with independent signal.

snapshots	20	30	40	50	60	70	80	90	100
DAS	0.342	0.257	0.241	0.241	0.209	0.196	0.184	0.180	0.178
IAA	0.148	0.128	0.125	0.112	0.110	0.105	0.100	0.102	0.102
SPICE	0.150	0.105	0.095	0.081	0.078	0.073	0.068	0.063	0.065
SPICE-ML	0.113	0.089	0.082	0.069	0.064	0.061	0.055	0.051	0.051

**Table 4:** RMSE vs. SNR with coherent signal.

SNR	0	2	4	6	8	10	12	14	16	18	20
DAS	0.625	0.617	0.593	0.589	0.589	0.590	0.588	0.582	0.579	0.581	0.579
IAA	0.376	0.322	0.273	0.233	0.210	0.190	0.179	0.166	0.159	0.155	0.150
SPICE	2.777	0.811	0.777	0.407	0.360	0.312	0.275	0.252	0.226	0.199	0.195
SPICE-ML	0.244	0.187	0.147	0.110	0.091	0.069	0.055	0.045	0.034	0.028	0.023

**Table 5:** RMSE vs. snapshots with coherent signal.

snapshots	20	30	40	50	60	70	80	90	100
DAS	0.637	0.626	0.617	0.613	0.617	0.606	0.603	0.609	0.609
IAA	0.163	0.143	0.135	0.130	0.124	0.124	0.117	0.112	0.117
SPICE	1.950	0.364	0.321	0.311	0.297	0.285	0.285	0.292	0.276
SPICE-ML	0.114	0.094	0.078	0.074	0.065	0.062	0.056	0.052	0.053
